# A Bibliometric Analysis of Healthcare Intervention-Related Studies Reporting Patient and Public Involvement and Engagement

**DOI:** 10.3390/healthcare13030305

**Published:** 2025-02-02

**Authors:** Wenze Lu, Yan Li, Jed Montayre, Mengqi Li, Ka Yan Ho, Jiaying Li, Janelle Yorke

**Affiliations:** School of Nursing, The Hong Kong Polytechnic University, Building GH, 11 Yuk Choi Road, Hung Hom, Kowloon, Hong Kong SAR 999077, China; chriswenze.lu@polyu.edu.hk (W.L.); yan-nursing.li@polyu.edu.hk (Y.L.); jed-ray.montayre@polyu.edu.hk (J.M.); kyeva.ho@polyu.edu.hk (K.Y.H.); jia-ying.li@polyu.edu.hk (J.L.)

**Keywords:** patient and public involvement and engagement, bibliometric review, healthcare, intervention, themes, trends

## Abstract

Background/Objectives: Patient and public involvement and engagement (PPIE) has gained global recognition as an innovative healthcare research practice. PPIE engages end-users throughout the research process, improving intervention effectiveness, resource efficiency, and user satisfaction. Despite its increasing inclusion in studies, comprehensive bibliometric reviews of healthcare intervention-related studies reporting PPIE are scarce. This study aims to conduct a bibliometric analysis of healthcare intervention-related studies reporting PPIE in recent decades to identify key worldwide bibliometric features, themes, and trends. Methods: The analysis includes 10,624 relevant English articles published in the Web of Science (WoS) Core Collection up to 26 November 2024. Search terms were selected based on PPIE conceptualization, interventional types, and related healthcare terms. Using WoS descriptive analysis and CiteSpace, we examined bibliometric features and identified major international themes and trends. Results: There has been a significant increase in the number of healthcare intervention-related studies reporting PPIE over the past five years, especially from the United States and the United Kingdom, with a recent rise in Asia. However, cross-national collaboration remains limited. Key research themes identified include “community participation”, “health equity”, “coronary heart disease”, “web-based patient empowerment”, “mental illness”, and “obesity prevention”, with growing interest in “mobile health” and “digital health”. Conclusions: This study provides a comprehensive and up-to-date overview of the bibliometric characteristics and evolving trends in healthcare intervention-related studies reporting PPIE. It highlights global regions with limited PPIE implementation, suggests pathways for further development, and identifies key research themes. The study offers researchers and practitioners valuable insights into tracking PPIE trends in healthcare interventions and fostering collaborations on evidence-based PPIE studies with leading scholars and institutions worldwide. Additionally, the findings drive innovations aimed at improving patient and public healthcare outcomes.

## 1. Introduction

Patient and public involvement and engagement (PPIE), consumer engagement, co-design, and co-production are often used interchangeably in healthcare research, reflecting the involvement of relevant individuals in planning, designing, and/or delivering research [1,2,3,4,5,6]. In this review, we will use PPIE to incorporate these different terms. PPIE represents a collaborative partnership between researchers and patients or the public. It refers to the involvement of patients and the public in key stages of the research process, including problem identification, study design, selection of outcome measures, execution of research, interpretation of results, evaluation of research outcomes, and dissemination of findings [7,8,9]. There is a well-established consensus that embedding PPIE in health-related research improves the relevance, quality, effectiveness, and impact of the research [3,6,9]. For healthcare interventions, PPIE has become increasingly recognized for its contributions to improving intervention resource utilization, relevance, acceptability, and efficacy [10,11,12], which has led to a substantial increase in related research publications. However, there remains a gap in a global and up-to-date review of the bibliometric profiles, themes, and historical development trends in healthcare intervention-related studies reporting PPIE. Understanding these bibliometric characteristics and trajectories is important for researchers. It helps them identify relevant focal points for future publications in healthcare intervention fields, seek collaborations across authors, institutions, and countries, and use PPIE in healthcare interventions for diverse populations. For practitioners, these research features can inform practical ideas and foster collaboration with appropriate scholars.

PPIE is increasingly being integrated into the development of healthcare services and the formulation of healthcare policies [13]. By aligning research with patient needs and public concerns, PPIE helps to direct the focus of healthcare services towards these critical issues [14]. Including patients and the public in the design of research methodologies improves both the practicality and the acceptability of the services proposed [6]. Insights from PPIE provide essential real-world perspectives that guide policy decisions and facilitate policy adaptations that meet practical implementation challenges and match patients’ and the public’s preferences [15]. The integration of diverse voices into research also increases the likelihood of gaining social support, thereby strengthening advocacy for policies derived from the research [13]. Furthermore, PPIE is considered a requirement for securing some research funding in the UK, with funding bodies like the National Institute for Health and Care Research (NIHR) mandating its inclusion in applications [4]. Applicants need to detail PPIE from a project’s design to its execution and impact evaluation on standard forms and specify budget allocations for this purpose [4]. Regarding healthcare interventions, PPIE helps shape the initial purpose, set priorities, and frame agendas during the planning stage of an intervention [2]. During a design stage, PPIE ensures that end-users’ preferences and needs are adequately considered and discussed for increasing the relevance, acceptability, and effectiveness of an intervention [10,16]. At the implementation stage, PPIE fosters a sense of ownership and empowerment among relevant stakeholders, driving higher levels of engagement and participation [16]. By encouraging patient and public participation in decision-making processes and providing customized education about interventions, healthcare researchers and professionals can align healthcare plans more closely with users’ preferences, thereby improving clinical outcomes and building long-term trust [17,18]. Ultimately, PPIE not only improves individual patient and public care but also influences broader practices, making them more responsive to the real-world complexities and needs of healthcare [17].

Earlier reviews on PPIE in healthcare primarily used systematic and narrative literature reviews [19,20,21]. These reviews contribute to the description and evaluation of specific questions related to the use of PPIE in healthcare [22,23]. However, there is a lack of a global overview on the research features and trends of studies reporting PPIE in healthcare interventions. A bibliometric review (BR) can complement previous review methods by offering a broader and more objective analysis, systematically evaluating bibliometric features including research areas, journals, authors, affiliations, keywords, themes, the literature, trends, and collaboration networks [24]. BR utilizes mathematical and statistical tools to visually and statistically analyze large-scale publications within a research field over the years, presenting foundational research components such as authors, institutions, journals, keywords, and references [24]. It also identifies the main research themes and developmental trends to provide a clear picture of the current status of the field and guide future studies [25,26].

## 2. Review Aim and Question

This study aimed to conduct a BR to elucidate the bibliometric characteristics and trends in healthcare intervention-related studies reporting PPIE from an international perspective to inform future research and practices. Applying a general BR approach to the existing literature, the specific review questions are as follows:

Review question 1 (RQ1): What are the yearly distributions of published articles, authors, journals, and influential authors/journals/references in healthcare intervention-related studies reporting PPIE?

Review question 2 (RQ2): What are the distributions of countries and their trends in healthcare intervention-related studies reporting PPIE?

Review question 3 (RQ3): What are the themes and their trends in healthcare intervention-related studies reporting PPIE?

We consider “intervention-related studies” to be those that focus on evaluating the effects and outcomes of specific strategies or programs designed to improve health, behavior, or social conditions [27]. The methodologies for healthcare interventions are diverse, including randomized controlled trials, clinical trials, therapeutic interventions, and other types [28,29]. Our focus is specifically on research that reports PPIE in healthcare interventions. It is not required that these studies employ PPIE as a primary intervention. This study followed the preliminary guidelines for reporting bibliometric reviews of biomedical literature (BIBLIO) [30] and the preferred reporting items for bibliometric analysis (PRIBA) in health and medicine-related studies [31] to structure each section of this review.

## 3. Materials and Methods

### 3.1. Database Selection

This study employed the Web of Science (WoS) as the sole database. In bibliometric research, the WoS database is highly regarded for its detailed and comprehensive record-keeping, particularly in medicine, health sciences, nursing, and healthcare [32]. It serves as a crucial bibliographic repository and is instrumental in research evaluations and bibliometric analysis [32]. While other databases are also valuable for review studies, the WoS distinguishes itself with its systematic approach, quality of publications, and advanced bibliometric capabilities including detailed indices and more accurate data extraction (e.g., author names and citation counts) [33,34].

### 3.2. Data Collection and Data Period

This study collected BR data from targeted sub-datasets within the Web of Science (WoS) Core Collection, including the Social Sciences Citation Index (SSCI), Science Citation Index Expanded (SCI-E), Emerging Sources Citation Index (ESCI), and Arts & Humanities Citation Index (A&HCI), known for containing high-quality and influential publications [33,35]. To create a comprehensive database for healthcare intervention-related studies reporting PPIE, the study did not set a start date and included all publications available up to 26 November 2024 (the date when the data were collected).

### 3.3. Search Strategy and Inclusion Criteria

**Figure 1:**
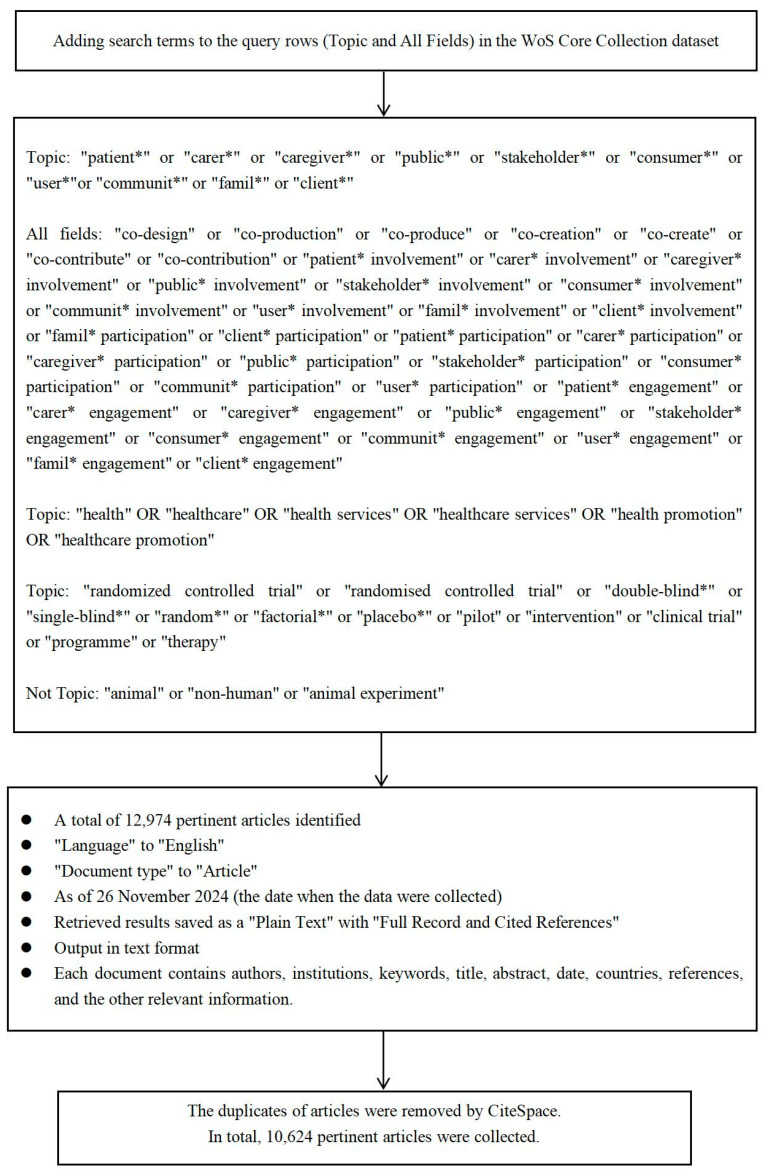
Search strategy and inclusion criteria of healthcare intervention-related studies reporting PPIE in the WoS.

To ensure the comprehensiveness and accuracy of data searching, the research team outlined a search strategy for systematic data collection based on prior PPIE studies and team discussions. We incorporated a range of terminologies linked to our conceptualization of PPIE, supplemented with synonyms from the Medical Subject Headings [36]. For instance, terms included not only “PPIE” but also “co-production”, “co-creation”, “co-design”, “consumer involvement”. “Patients” were defined broadly to include patients, carers, and users of health and social care services, along with their advocates; “Public” referred to all other potential service recipients and stakeholders [37]. Non-human and animal studies were excluded. Keywords relating to involvement and engagement were entered into the WoS “all field” search in case terms like “patient involvement” were not explicit in the title or abstract [37,38]. The search terms used to identify “intervention-related studies” included types of interventions referenced in prior research [27,28,29], such as “randomized controlled trial”, “clinical trial”, “factorial”, “placebo”, and “therapy”. This review is limited to English healthcare intervention-related studies that have reported PPIE and are published in the WoS. The decision to focus on the WoS was based on its rigorous indexing standards, comprehensive coverage of high-quality, peer-reviewed research, and advanced bibliometric tools that facilitate reliable and systematic analyses [32,33,34]. While it is acknowledged that other databases, such as Scopus or PubMed, could provide additional insights, the WoS was selected for its multidisciplinary scope, robust citation metrics, and proven reliability in bibliometric research. However, this selection may inherently exclude relevant studies indexed in other databases, which is recognized as a limitation of this review. Additionally, the research focus is not on studies exclusively about interventions on PPIE but rather on those where terms related to both interventions and PPIE are identified. The search strategy and inclusion criteria are shown in Figure 1.

### 3.4. Data Analysis

To address the review questions, we employed a bibliometric analysis of WoS and CiteSpace. CiteSpace 6.3.R1, an updated version of the CiteSpace series tool particularly useful for BR, was selected for this study. The bibliometric analysis of WoS is frequently employed to systematically examine foundational research characteristics of publications indexed in WoS, such as the number of yearly publications, authors, countries, institutions, and journals [33]. CiteSpace was specifically selected for its academic popularity and advanced capabilities in visualizing internal relationships, identifying emerging trends, and analyzing thematic patterns within diverse research areas [39]. Its user-friendly interface, combined with its professionalism and diverse bibliometric functions, makes it well suited for conducting in-depth bibliometric evaluations [33,40]. Both tools have been extensively utilized in bibliometric review studies across disciplines [33,39,40].

After removing duplicates of OMC articles using CiteSpace, we found that the earliest articles in our review emerged in 1991, totaling six from that year, and thus set the main parameters in CiteSpace as follows: time slicing (January 1991–November 2024), years per slice (1), term source (title, abstract, author, keywords, and keywords plus), node type (author, institution, country, keyword, source, and reference), selection criteria (top 10% of most-cited or occurred items from each slice), g-index (k = 25), pruning (pathfinder, pruning the merged network), and visualization (cluster viewstatic, showing merged network). The specific data analysis methods used to identify each bibliometric feature are shown in Table 1.

## 4. Results

This study conducted a BR of studies related to healthcare interventions reporting on PPIE, sourced from the WoS Core Collection, over recent decades. The review exclusively considered English-language articles focused on healthcare interventions that reported PPIE and were published in the WoS. The scope of this review extends beyond studies solely dedicated to interventions on PPIE to include those studies where both interventions and PPIE-related terms are identified. Our systematic data collection strategy was meticulously outlined, incorporating a broad spectrum of terminologies associated with our conceptualization of PPIE. This was further enriched with PPIE synonyms from the MeSH, methods employed in interventional studies, and various healthcare-related terms.

### 4.1. Yearly Published Articles, Authors, Influential Authors, and Journals

#### 4.1.1. Yearly Distribution of Published Articles, Journals, and Authors

**Figure 2:**
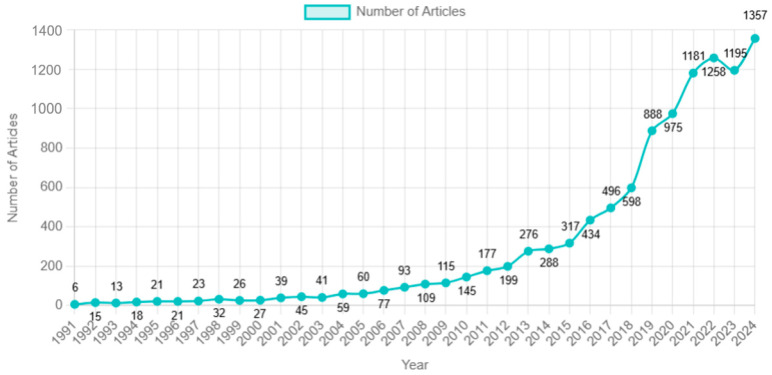
Yearly distribution of published research articles.

As shown in Figure 2, PPIE being referred to in healthcare intervention articles began appearing in 1991, with a gradual increase over the following two decades. A marked rise is evident from 2018 onwards. According to Table 2, the top journals publishing these articles include *BMJ Open*, *BMC Health Services Research*, *Health Expectations*, and other high-impact journals. Most of them are open access from medical fields. The most prolific authors by number of publications are Wells Kenneth, Joseph Tucker, Lovell Karina, Mullins Daniel, Montori Victor Manuel, and Chaboyer Wendy.

#### 4.1.2. Distribution of Influential Authors/References/Journals

The top ten authors, based on citation counts, are listed in Table 3. These authors have made significant contributions to healthcare intervention-related studies reporting PPIE and are considered influential in this area. Among these, authors representing the World Health Organization received the highest citation count, followed by Vittoria Braun (Charité-Universitätsmedizin Berlin, Germany), Susan Michie (University College London, UK), Albert Bandura (Stanford University, USA), and Trisha Greenhalgh (University of Oxford, UK).

Similarly, the top articles based on citation counts were identified and subsequently verified by the authors (see Table 4). These articles were not original interventional studies employing PPIE to change subjects’ outcomes. Instead, they were identified based on the presence of terms related to both interventions and PPIE within the search system. These articles offer frameworks and guidelines that contribute to the understanding of the evolution and importance of PPIE in healthcare intervention-related studies [13,14,15,46]. They also demonstrate various formats for implementing PPIE in the development and evaluation of interventions [2,21,47], and discuss practical applications of PPIE and its impact in diverse settings [16,48,49].

Table 5 also lists the top ten journals and their citation counts. It was found that *PLOS ONE* had the highest citation count (2791) in the study field, followed by *BMJ* (2668), *the Lancet* (2656), and *JAMA* (2379).

#### 4.1.3. Distribution of Author Collaboration

**Figure 3:**
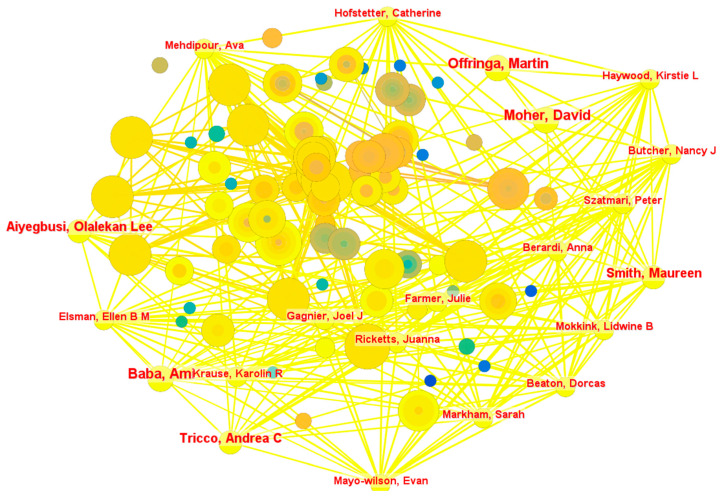
Author collaboration network. Note: This figure depicts the collaborative relationships among authors contributing to PPIE healthcare intervention research. Each node represents an author, with the node size proportional to the number of publications authored. Links between nodes signify co-authorship relationships, where thicker and shorter links indicate stronger and more frequent collaboration. The listed authors reflect their influential roles in fostering international collaboration in PPIE healthcare intervention research and serve as key connectors, bridging multiple clusters and facilitating cross-disciplinary research efforts.

The author collaboration network is illustrated in Figure 3. The analysis identified 1273 nodes (authors) and 1853 collaboration links and reveals several prominent co-authorship clusters, indicating concentrated efforts among specific groups of researchers to advance knowledge in healthcare interventions reporting PPIE. Notable leaders within these clusters include Beaton Dorcas, who maintained 19 collaborative links, and Baba Ami, Smith Maureen, Hofstetter Catherine, Markham Sarah, Butcher Nancy, Elsman Ellen, Ricketts Juanna, Haywood Kirstie, and Farmer Julie, each with 18 collaborative links. These authors occupy central positions within the network, indicating their roles in driving collaborative research within this domain.

A closer examination of the network reveals an uneven distribution of collaboration, with significant clustering around key individuals and groups. It suggests that while certain authors are interconnected, other areas of the network exhibit weaker collaborative ties. Such disparities highlight opportunities for fostering broader interdisciplinary engagement and integrating less-connected researchers into more active networks. The reliance on central authors, while beneficial for facilitating collaboration and knowledge exchange, may also pose challenges, such as bottlenecks in research innovation or over-dependence on a few researchers to sustain international collaborations in the field. Clusters with high connectivity demonstrate the potential to accelerate the exchange of ideas, drive innovation, and contribute to a more cohesive body of knowledge. A more active and even collaborative network is needed for a more inclusive and diverse research community to foster greater equity in the generation and dissemination of knowledge.

### 4.2. Countries

#### 4.2.1. Distribution of Countries and Country Collaboration

**Figure 4:**
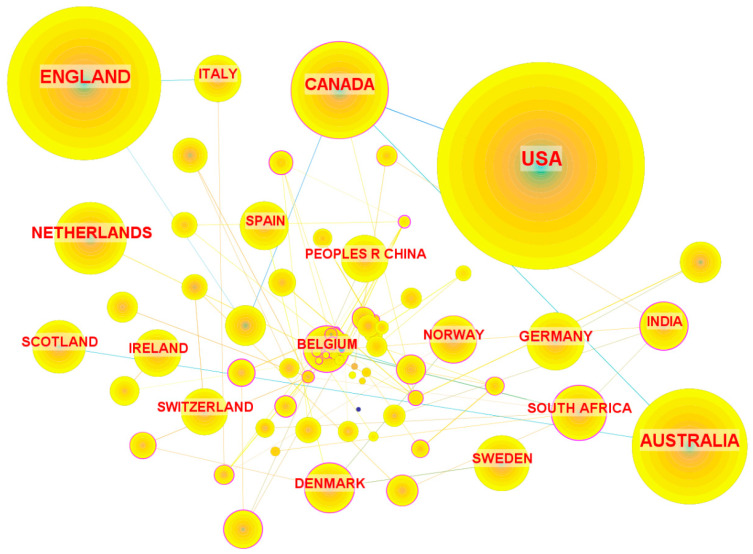
Country collaborative network. Note: This figure illustrates the collaborative relationships between countries involved in PPIE healthcare intervention research. Each node represents a country/region, with the node size corresponding to the volume of publications produced by that country/region. Links between nodes indicate collaborative relationships. The USA, England, Canada, and Australia are prominent nodes, highlighting their central and active roles in driving international collaborations. Smaller nodes, such as Belgium, Denmark, and Switzerland, indicate contributions from countries with fewer publications but are important collaborative roles within the network.

The distribution of countries is shown in Table 6. The majority of these studies originated from the USA (40.73%), England (22.50%), Australia (12.82%), and Canada (9.36%). The collaborative network among countries, shown in Figure 4, comprises 74 nodes and 2082 links. Lebanon leads with the highest number of collaborative links (*n* = 9), followed by Argentina, Chile, and South Africa with 6 each, and Canada, Singapore, Denmark, India, Belgium, and Poland with 4 each. Notably, Lebanon, South Africa, and India demonstrated betweenness centrality values exceeding 0.5, followed by Belgium, Argentina, Chile, Singapore, Nepal, and Slovenia. The network highlights a dynamic and influential collaboration landscape in global healthcare intervention research related to PPIE.

**Figure 5:**
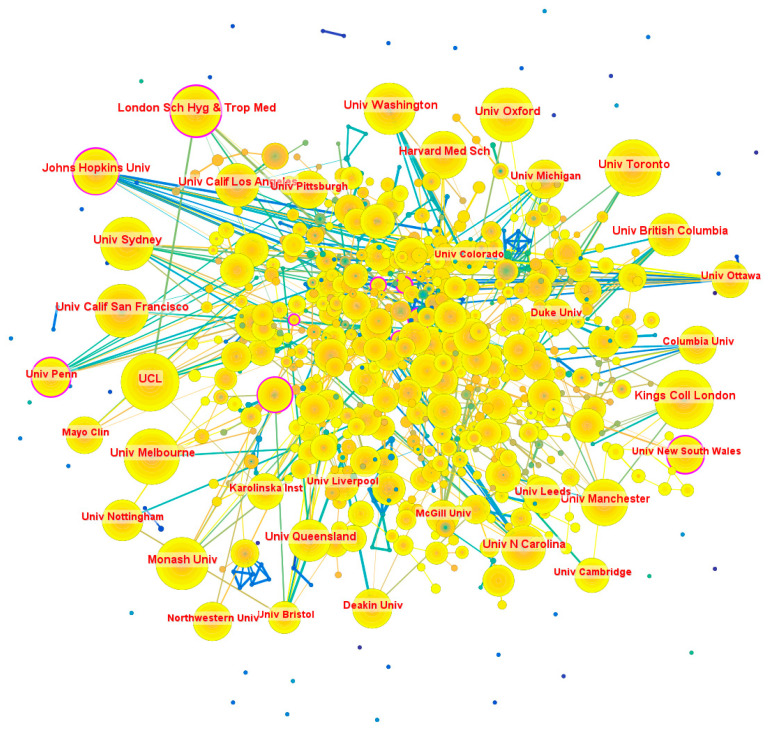
Institutional collaboration network. Note: This figure illustrates the collaborative relationships between institutions involved in PPIE healthcare intervention research. Each node represents an institution, with node size proportional to the institution’s publication output in the dataset. The links between nodes indicate collaborative relationships, where thicker and shorter links represent stronger and closer collaborations. Network clustering patterns reveal distinct groups of institutions with denser interconnections, which likely arise from factors such as geographic proximity, focused research agendas, or longstanding collaborative networks. The patterns highlight the varying levels of integration among institutions, with some forming strong collaborative hubs while others remain more peripheral.

Regarding the distribution of institutions, the most productive institutions are predominantly based in the USA and the UK (see Table 7). The institutional collaboration network, consisting of 863 nodes and 969 links, revealed an uneven distribution (see Figure 5). Notable institutions include Johns Hopkins University (US) with 15 links, the University of Ottawa (Canada) and Harvard University (US) with 14 links each, and the University of Sydney (Australia) with 11 links.

#### 4.2.2. Trends of Countries/Regions

**Figure 6:**
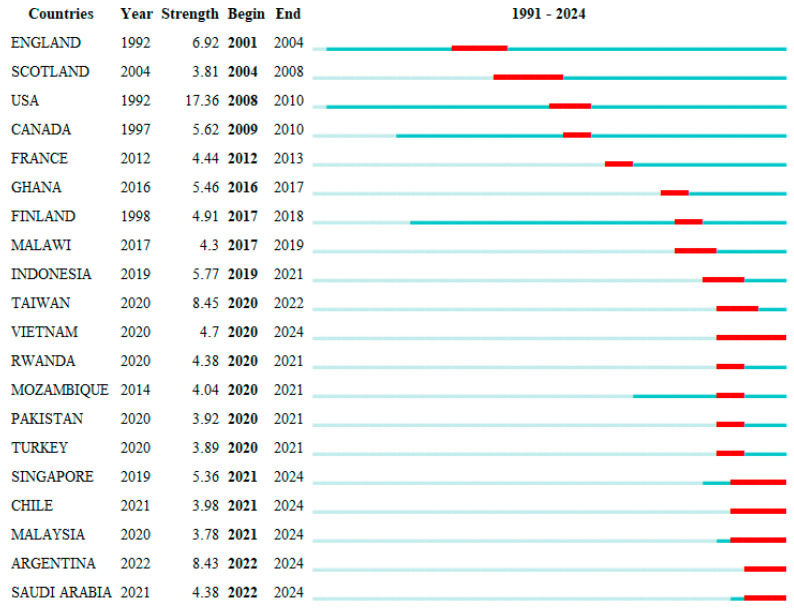
Top 20 countries/regions with the strongest citation bursts. Note: Light blue bars represent the period before relevant articles from a country/region were published, while dark blue bars indicate the onset of publication. Red segments mark the start and end of a citation burst period.

Figure 6 identifies the top 20 countries/regions exhibiting the strongest citation bursts. The USA displayed the strongest citation burst (17.36), indicating that PPIE healthcare intervention research from the USA attracted the most substantial global attention and was cited the highest between 2008 and 2010. Other longer periods of PPIE healthcare intervention research development were observed in Scotland from 2004 to 2008 and Vietnam from 2020 to 2024. From 2021 to 2024, Asian countries such as Singapore, Malaysia, and Saudi Arabia experienced a surge in PPIE healthcare intervention research, indicating the growing prominence of Asia in the global research landscape in this field. The expanding contributions from Asia also demonstrate its increasing influence on recent global PPIE healthcare intervention research directions and highlight the potential need for cross-regional collaboration to translate regional outcomes into broader healthcare progress.

### 4.3. Themes

#### Identification of Themes

The Web of Science (WoS) database identified the top ten research areas of healthcare intervention-related studies reporting PPIE (see Table 8). These articles span diverse disciplines, demonstrating the interdisciplinary nature of healthcare intervention research reporting PPIE.

These research areas represent a broad and overarching set of disciplines or categories as recognized by the WoS classification system. Building upon this initial analysis, a subsequent theme investigation was conducted using the article title, keyword and abstract extraction, and clustering function in CiteSpace. This allowed for the identification of more focused, specific, and subject-matter-oriented research themes. The top ten distinct clusters were identified and are presented in Table 9, including “community participation”, “health equity”, “coronary heart disease”, “web-based patient empowerment”, “mental illness”, “obesity prevention”, “intervention mapping”, “healthy behavior”, “reporting guidance”, and “special populations network”. Cluster size reflects similar article counts, while homogeneity is measured by a Silhouette score, where a score close to 1 signifies high similarity within the cluster [33]. A mean score above or nearly equal to 0.5 is deemed acceptable [50]. Table 9 also details key terms and the mean publication year for each cluster, highlighting “community participation”, “obesity prevention”, and “special populations network” as the earliest studied themes. A representative article for each cluster is highlighted.

### 4.4. Trends of Themes

**Figure 7:**
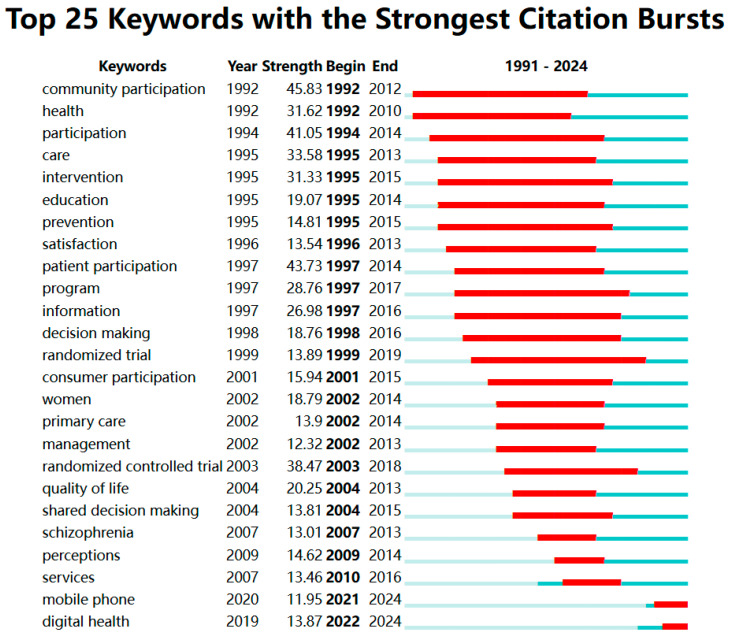
Top 25 keywords with the strongest citation bursts. Note: Light blue bars represent the period before the keywords appeared, while dark blue bars indicate the onset of keyword appearance. Red segments mark the start and end of a keyword citation burst period.

Figure 7 presents the top 25 keywords with the most substantial citation bursts as identified by CiteSpace, suggesting these terms represent the dominant themes in PPIE healthcare interventional studies during the specified years. The keyword “community participation” exhibited the highest burst strength at 45.83, reflecting its centrality and enduring influence in the field. The prominence of keywords like “community participation”, “patient participation”, and “consumer participation” demonstrates the foundational role of participatory approaches in shaping PPIE healthcare research and practices. It also reflects a growing recognition of the importance of engaging communities, patients, and consumers as active stakeholders in healthcare decision-making and intervention design. Their high burst strengths and extended periods of influence suggest that they have been well integrated into the research.

Other significant keywords, such as “randomized trial” (1999–2019), “prevention” (1995–2015), “intervention” (1995–2015), and “education” (1995–2014), demonstrated longer burst cycles which indicate their sustained relevance over extended periods. They may also point to a sustained emphasis on rigorous methodologies, preventive approaches, and educational strategies to improve healthcare outcomes. More recent keywords with shorter burst periods include “mobile phone” (2021–2024) and “digital health” (2022–2024), reflecting emerging trends and the evolving focus on technology-driven healthcare interventions that utilize PPIE. The shorter burst periods of these keywords may also signal the rapid pace of innovation and adoption in digital health, as well as the responsiveness of the field to technological advancements.

Overall, the thematic evolution of keywords highlights the dynamic and multidimensional nature of research within PPIE healthcare interventions. The coexistence of long-standing themes with emerging trends suggests that while the field continues to build on established foundations, it is also highly adaptive to new challenges and opportunities. The growing focus on “digital health” and “mobile phone” interventions may reflect efforts to address global health challenges, such as disparities in access to care, by leveraging scalable and cost-effective solutions. Meanwhile, the enduring relevance of participatory approaches reinforces the imperative to center research around the needs and perspectives of patients, consumers, and communities.

## 5. Discussion

### 5.1. Increasing Publications and Collaborative Challenges

To our knowledge, our BR is the first one to describe bibliometric characteristics and evolving trends in healthcare intervention-related studies reporting PPIE in recent decades. Our results indicate that globally implemented studies using PPIE have grown considerably over the past two decades, with a particularly notable increase in the last five years. Particularly in the past three years, there has been a significant increase in the Asian region. The growing emphasis on PPIE in healthcare intervention-related studies may be due to its increasing recognition by researchers and strong advocacy from research organizations such as the UK Medical Research Council and NIHR [2,4,13]. International guidelines such as those from the Declaration of Helsinki and the European Clinical Trials Regulation continually highlight the importance of PPIE in healthcare [58,59]. These efforts collectively promote a transparent, respectful, and inclusive research process, advocating for the integration of patient and public insights throughout healthcare interventions.

Our results suggest that most authors work independently or in pairs within the same organization. The most prolific, influential, and actively collaborating authors are distinct from each other. Most collaborating institutions are within the same country, highlighting a predominance of internal over cross-national collaboration and a lack of stable international partnerships. Cross-national collaborative links in PPIE healthcare intervention-related studies are sparse, with the most productive countries demonstrating less active collaboration with other nations in this field. Cross-national cooperation faces challenges such as language and cultural barriers, misinterpretations, and data security concerns [60,61]. Geographical limitations also pose obstacles, especially when involving multiple clinics or health organizations across extensive regions [62]. Socioeconomic and demographic variations further influence cross-national practices in healthcare interventions [63]. Additionally, patients from different geographical regions have varying needs and perspectives, complicating the development of universally applicable healthcare interventions. Countries or regions with less resources to implement PPIE in healthcare interventions may also present challenges for cross-national cooperation [7,9]. Limited financial resources, inadequate infrastructure, and restricted access to technology may exacerbate communication barriers and complicate the harmonization of healthcare practices across regions [13,61]. Those countries or regions often lack reliable mechanisms for secure data sharing and effective coordination among stakeholders [9,62]. The absence of context-specific frameworks in low-resource environments makes it difficult to implement universally applicable healthcare interventions using PPIE [46]. Despite these challenges, international co-authoring and institutional collaboration hold the potential to improve research creativity, facilitate worldwide knowledge sharing, and enhance the quality of research outcomes [33]. International collaborative efforts are also beneficial for balancing research resources and fostering the equitable development of PPIE in healthcare interventions across various regions [2].

Further research into overcoming these barriers and promoting more active global collaborations is highly encouraged. It is recommended from an actionable perspective that international collaboration in PPIE healthcare intervention research be strengthened by establishing global frameworks that promote equitable resource sharing, inclusive governance structures, and open access platforms for data and knowledge exchange. Interdisciplinary networks should be developed to foster trust, mutual learning, and the co-creation of research agendas across regions. For under-represented regions, targeted global funding mechanisms are needed to address systemic disparities through investments in interventional research programs utilizing PPIE, training programs for PPIE implementation, and sustainable access to digital tools. Furthermore, establishing monitoring and evaluation mechanisms to assess the impact of collaborative PPIE initiatives would enhance accountability, support continuous improvement, and ensure the long-term sustainability of international collaborations.

### 5.2. Leading and Emerging Countries

For the most productive countries, our results indicate that the USA and the UK are leading with high numbers of publications, contributing authors, influential authors, and institutions. Key authors, such as Albert Bandura in the USA and Susan Michie and Trisha Greenhalgh in the UK, have substantially contributed to these studies by promoting patient autonomy, informed choice, and the value of patients’ and the public’s preferences [21,64,65]. Major organizations like the UK’s Medical Research Council and the National Institute for Health and Care Research, along with the USA’s Agency for Healthcare Research and Quality and the Patient-Centered Outcomes Research Institute, collectively support the use of PPIE in research [66,67]. Researchers are encouraged to adopt advanced PPIE techniques from these leaders and seek collaborative opportunities for international studies.

For the most active countries, recently, Asian countries, including Singapore, Malaysia, and Saudi Arabia, have been notably identified, likely driven by increased healthcare funding, supportive government policies, and growing recognition of PPIE’s value in healthcare interventions [68,69]. For instance, Saudi Arabia’s Vision 2030 aims to improve health and quality of life, enhance healthcare delivery and accountability, and increase the value of care by managing costs and improving outcomes [70]. These initiatives highlight PPIE’s potential to effectively utilize collective knowledge and resources to tackle complex health challenges. In Singapore, the focus on PPIE in healthcare research is influenced by global trends, demographic shifts, and practical benefits [68]. Inspired by successful PPIE cases in Western countries, Singaporean researchers are striving to improve the relevance and effectiveness of healthcare interventions. PPIE in Singapore is expected to improve research recruitment and retention, shift ethical considerations by treating participants as partners, and respond to the challenges of an aging population, ultimately optimizing the impact and reducing waste in healthcare interventions [68,71]. However, China was notably absent from the list of productive and active countries in this study despite being one of the most prolific countries in overall research publication and having the largest population globally [72]. Investigating PPIE in China, with its vast research output and huge population, may provide unique insights into the use of PPIE across varied demographic and cultural contexts, thereby enriching global healthcare intervention practices.

### 5.3. Key Research Themes and Trends

Our results also show the top ten research themes identified, among which “coronary heart disease”, “mental illness”, and “obesity prevention” are recognized as key health issues. This suggests that PPIE was much more frequently studied and examined in these contexts compared to others. Coronary heart disease is complex and chronic, often requiring long-term management and lifestyle changes. PPIE improves patient adherence by aligning interventions with patients’ values, preferences, and conditions, while ensuring patient-centered, relevant, and applicable strategies to promote prevention, support lifestyle modifications, and manage the disease [51]. Mental health interventions often require a high degree of personalization to be effective [53,73]. PPIE allows for the incorporation of patient experiences and preferences, which is crucial for tailoring interventions to address the unique needs of individuals with mental illnesses. Involving patients in the design and delivery of mental health services helps reduce stigma by normalizing discussions around mental health and demonstrating that patient input is valued and essential [74]. Mental health services benefit from peer support structures, and PPIE facilitates the development of peer-led interventions which have been shown to improve engagement and outcomes in mental health care [75]. PPIE is also frequently mentioned in the context of obesity prevention. This is likely because addressing this issue often requires interventions that engage not just the child, but their entire family and community [76,77]. PPIE enables the inclusion of these critical stakeholders in developing strategies that are culturally sensitive and practically feasible, thereby improving the overall effectiveness of the interventions [78]. Effective obesity interventions rely on sustained behavioral changes, which are more likely to occur when interventions are co-designed with input from children and their families in a realistic and supportive manner [79].

“Health equity” was also highlighted as a key theme. It means eliminating avoidable differences between groups and is important for making healthcare interventions accessible and suited to the needs and preferences of the target populations [80]. PPIE aims to involve diverse populations, particularly ethnic minorities, to address the needs of under-represented and disadvantaged groups and advance health equity [52]. However, challenges include lower participation rates among disadvantaged patients and potential oversight of ethnic minorities in recruitment [52,80]. In low-resource settings, the implementation of PPIE also faces obstacles due to systemic inequities, such as insufficient funding, limited healthcare access, and social exclusion of vulnerable populations [45,46,52]. The lack of standardized guidelines and regulatory frameworks for resource-constrained settings leads to inconsistencies in PPIE practices [46]. There is a need for comprehensive regulations and guidelines to standardize PPIE practices across regions and disciplines, especially in settings with limited resources. Additionally, the study indicated a trend in healthcare intervention studies reporting PPIE toward “digital health” and “mobile health” topics, reflecting the growing reliance on digital tools to involve and engage patients and the public. Digital platforms offer unique capabilities that traditional methods lack, such as the ability to provide real-time interaction and feedback, which is essential for continuous involvement and engagement [75]. They can also reduce barriers to participation and enable patients to engage with their healthcare on their own terms [81]. This flexibility is particularly valuable for individuals with busy schedules, mobility issues, or those living in remote areas. Digital data-driven approaches enable the customization of healthcare interventions to better meet the specific needs of each involved individual [82]. Additionally, digital platforms facilitate comprehensive data collection, which can be used to monitor interventional progress, identify trends, and make adjustments to interventions [83].

### 5.4. Limitations

Limitations arise from focusing on high-quality articles published in English and indexed in the WoS, which may not represent findings from non-English publications or those excluded by the WoS. We utilized BR techniques to present a clear and comprehensive bibliometric overview by analyzing a large dataset through CiteSpace. However, the extensive dataset and specific functionalities of CiteSpace might limit in-depth qualitative analyses and interpretations from diverse perspectives, such as PPIE patterns in different studies and the specific applications of PPIE across various countries. They may also limit the clarity of visualization display functions, making the figures appear unclear for more comprehensive analyses (e.g., dual map overlay). Additionally, our search strategy for identifying healthcare intervention-related studies reporting PPIE was based on our definition and understanding of PPIE and interventional studies. Using different search terms might have produced different results. Future studies could address these limitations by diversifying the databases used, such as incorporating Scopus, PubMed, or regional databases, to capture a broader range of publications, including non-English and region-specific studies that may provide unique perspectives on PPIE practices. Additionally, integrating qualitative methodologies, such as thematic analysis or case studies, could offer deeper insights into the contextual factors influencing PPIE implementation and the specific challenges faced in different healthcare systems and cultural settings. Employing iterative and inclusive search strategies, such as refining search terms through stakeholder consultation or pilot searches, would help account for variability in how PPIE and interventional studies are defined and reported across disciplines and regions.

## 6. Conclusions

This paper addresses a research gap by presenting a global and up-to-date BR of healthcare intervention-related studies reporting PPIE. It identifies key bibliometric features, themes, and trends within the field, providing researchers and healthcare professionals with a clearer understanding of the trajectory of relevant studies. The paper highlights potential areas for future research and practical application, offering guidance for advancing the field. Researchers can refer to this study and its findings to identify relevant focal points for future publications, seek collaborations across authors, institutions, and countries, and properly use PPIE in different research stages. Given the recent increase in research outputs from Asia, where fewer countries have historically been identified as productive in PPIE-related research, it is recommended to develop strategies or policies to support Asian researchers in actively engaging, implementing, and promoting PPIE. Frontline healthcare professionals are expected to utilize PPIE properly to optimize the development, design, and implementation of healthcare interventions. For example, given the emerging trend of digital and mobile health, practitioners can leverage digital tools such as mobile health apps, virtual platforms, and telehealth systems to facilitate more effective and inclusive PPIE. These tools can streamline communication, improve accessibility to underserved populations, and provide real-time feedback mechanisms, enabling practitioners to capture diverse patient perspectives more systematically and efficiently. For instance, mobile health apps can be used to collect patient-reported outcomes or preferences, while virtual platforms can host consultations or focus groups that accommodate participants from geographically remote areas. Policymakers can create enabling environments that support the adoption of PPIE in digital health, which may include embedding PPIE utilization into regional digital health strategies and allocating targeted funding to incentivize PPIE-driven projects. They can also establish training programs and technical support systems to empower healthcare practitioners to use digital tools effectively, while promoting data security and upholding ethical standards in PPIE activities. Lastly, this study offers healthcare professionals and researchers opportunities to collaborate with prominent scholars in developing evidence-based findings for using PPIE in research and practices related to healthcare interventions.

## Figures and Tables

**Table 1:** Data analysis methods for each bibliometric feature.

Bibliometric Features	Analysis Used	Purpose
Yearly Publications, Authors, Journals, and Countries	WoS bibliometric and descriptive analysis function	To identify each distribution in past decades
Influential Authors/Journals/References	Co-citation analysis through CiteSpace [41,42]	To identify citation counts for academic influence evaluation
Collaborations Among Authors And Countries	Network visualization through CiteSpace [33]	To identify productive authors and countries in global collaborations
	Betweenness centrality analysis through CiteSpace [43]	To identify active cross-country collaboration and country influence
Thematic Analysis	Co-citation cluster analysis through CiteSpace [44,45]	To measure thematic similarities between articles, with titles, keywords, and abstracts analyzed for theme identification
Thematic Labeling	Log-likelihood ratio method through CiteSpace [26]	To provide precise thematic labeling by derivation from article titles, keywords, and abstracts
Trend Analysis for Countries	Citation bursts analysis through CiteSpace [33]	To reveal past developmental trends in country participation based on periods of significantly increased research outputs and to identify recently active countries
Trend Analysis for Themes	Keyword bursts (using both author keywords and keywords plus from WoS) through CiteSpace [45]	To identify evolving thematic trends and areas of growing academic interest

**Table 2:** Top ten journals and authors regarding the number of published research articles.

Ranking	Journal Title	No. of Articles (%)	Authors	No. of Articles (%)
1	BMJ Open	417 (3.93)	Wells Kenneth	32 (0.30)
2	BMC Health Services Research	244 (2.30)	Tucker Joseph	24 (0.23)
3	Health Expectations	233 (2.19)	Lovell Karin	23 (0.22)
4	PLOS ONE	217 (2.04)	Mullins Daniel	21 (0.20)
5	BMC Public Health	204 (1.92)	Montori Victor Manuel	21 (0.20)
6	Journal of Medical Internet Research	175 (1.65)	Chaboyer Wendy	21 (0.20)
7	International Journal of Environmental Research and Public Health	156 (1.47)	Lucy Yardley	20 (0.19)
8	Trials	141 (1.33)	Chung Bowen	19 (0.18)
9	JMIR Formative Research	138 (1.30)	Tang Lingqi	19 (0.18)
10	Patient education and counseling	129 (1.21)	Miranda Jeanne/Petrou Stavros/Mcelfish Pearl/Staniszewska Sophie/Bower Peter	18 (0.17)

**Table 3:** Top ten authors based on author co-citation network analysis.

Rank	Number of Citations	Author
1	1727	World Health Organization
2	1085	Braun Vittoria
3	498	Susan Michie
4	392	Albert Bandura
5	389	Trisha Greenhalgh
6	372	Tong Allison
7	318	Kroenke Kurt
8	309	Laura Damschroder
9	303	Glasgow Robert
10	284	Patton Michael

**Table 4:** Top ten references based on reference co-citation network analysis.

Rank	Number of Citations	Article	Journal
1	3502	Moore et al. [15]	BMJ
2	2393	Skivington et al. [13]	BMJ
3	1954	Stacey et al. [49]	Cochrane Database of Systematic Reviews
4	809	Brett et al. [16]	Health Expectations
5	755	Alicia et al. [14]	BMJ Open
6	604	Perski et al. [47]	Translational Behavioral Medicine
7	585	Yardley et al. [48]	American Journal of Preventive Medicine
8	575	Staniszewska et al. [46]	BMJ
9	515	Slattery et al. [2]	Health Research Policy and Systems
10	370	Greenhalgh et al. [21]	Health Expectations

**Table 5:** Top ten journals based on journal co-citation network analysis.

Rank	Number of Citations	Journals
1	2791	Plos One
2	2668	BMJ
3	2656	Lancet
4	2379	JAMA
5	2181	Social Science & Medicine
6	2019	BMJ Open
7	1886	BMC Health Services Research
8	1824	BMC Public Health
9	1716	American Journal of Public Health
10	1643	Journal of General Internal Medicine

**Table 6:** Top ten countries regarding the number of published research articles and centrality values.

Rank by No. of Articles	Country	No. of Articles (%)	Rank by Betweenness Centrality Value	Country/ No. of Articles	Betweenness Centrality Value *
1	USA	4327 (40.73)	1	Lebanon (12)	0.89
2	England	2390 (22.50)	2	South Africa (300)	0.69
3	Australia	1362 (12.82)	3	India (225)	0.51
4	Canada	994 (9.36)	4	Portugal (72)	0.50
5	Netherlands	557 (5.24)	5	Belgium (210)	0.49
6	Germany	357 (3.36)	6	Argentina (20)	0.46
7	Sweden	324 (3.05)	7	Chile (13)	0.45
8	South Africa	318 (2.99)	8	Singapore (55)	0.43
9	Scotland	311 (2.93)	9	Nepal (25)	0.43
10	Denmark	268 (2.52)	10	Slovenia (4)	0.39

* Note: Betweenness centrality is a network analysis metric that measures the extent to which a node (e.g., a country) acts as a bridge between other nodes by lying on the shortest paths connecting them [43]. This metric can indicate the structural role of a node in connecting different clusters of research activity for international collaboration [39]. Countries with high betweenness centrality values are positioned as key intermediaries, facilitating connections across otherwise disconnected parts of the research network [33].

**Table 7:** Top ten institutions regarding the number of published research articles.

Ranking	Institution	No. of Articles (%)	Country
1	University of London	846 (7.96)	UK
2	University of California System	551 (5.19)	US
3	Harvard University	361 (3.40)	US
4	University College London	325 (3.06)	UK
5	University of Toronto	295 (2.78)	Canada
6	Kings College London	293 (2.76)	UK
7	Johns Hopkins University	288 (2.71)	US
8	University of Sydney	272 (2.56)	Australia
9	University of North Carolina	267 (2.51)	US
10	US Department of Veterans Affairs	258 (2.43)	US

**Table 8:** Top ten research areas regarding the number of published research articles identified by the WoS.

Ranking	Research Area	No. of Articles (%)
1	Public Environmental Occupation Health	2802 (26.38)
2	Health Care Sciences Services	2522 (23.74)
3	General Internal Medicine	1153 (10.85)
4	Psychiatry	757 (7.13)
5	Medical Informatics	751 (7.07)
6	Psychology	711 (6.69)
7	Nursing	547 (5.15)
8	Rehabilitation	504 (4.74)
9	Research Experimental Medicine	379 (3.57)
10	Environmental Sciences Ecology	300 (2.82)

**Table 9:** Theme identification by titles, keywords, and abstracts in healthcare intervention-related studies reporting PPIE.

Cluster	Size	Silhouette	Label (Log-Likelihood Ratio) (LLR)	Average Year	Top Terms	Representative Article
1	89	0.898	Community participation	2004	impact; outcome; program; prevention; quality	Arnett et al. (2019). 2019 ACC/AHA guideline on the primary prevention of cardiovascular disease: a report of the American College of Cardiology/American Heart Association Task Force on Clinical Practice Guidelines [51].
2	50	0.877	Health equity	2019	mental health; community engagement; community-based participatory research	Simon et al. (2020). Patient and family engaged care: an essential element of health equity. NAM perspectives, 2020 [52].
3	49	0.859	Coronary heart disease	2017	physical activity; adherence; older adults; trial; social support	Arnett et al. (2019). 2019 ACC/AHA guideline on the primary prevention of cardiovascular disease: a report of the American College of Cardiology/American Heart Association Task Force on Clinical Practice Guidelines [51].
4	48	0.842	Web-based patient empowerment	2018	involvement; health literacy; individuals; internet	Geerling et al. (2022). A web-based positive psychology app for patients with bipolar disorder: Development study [53].
5	48	0.924	Mental illness	2016	impact; outcome; people; services; schizophrenia	Granlund et al. (2021). Definitions and operationalization of mental health problems, wellbeing and participation constructs in children with NDD: distinctions and clarifications [54].
6	47	0.901	Obesity prevention	2005	care; intervention; children; program; qualitative research	Whelan et al. (2022). Reflexive evidence and systems interventions to prevention obesity and non-communicable disease (RESPOND): protocol and baseline outcomes for a stepped-wedge cluster-randomised prevention trial [55].
7	46	0.92	Intervention mapping	2014	participation; risk; depression; barriers; adults	Eaton et al. (2021). Training Peers to Ease Hospital Discharge: A Community–Clinical Partnership in Complex HIV Care [56].
8	46	0.879	Healthy behavior	2018	randomized controlled trial; digital health; mobile phone; cognitive behavioral therapy	Pozuelo et al. (2023). User-Centered Design of a Gamified Mental Health App for Adolescents in Sub-Saharan Africa: Multicycle Usability Testing Study [57].
9	46	0.906	Reporting guidance	2018	people; services; implementation science; technology; life	Husereau et al. (2022). Consolidated Health Economic Evaluation Reporting Standards 2022 (CHEERS 2022) statement: updated reporting guidance for health economic evaluations [58].
10	40	0.883	Special populations network	2010	health; prevention; quality; primary care; community participation	Eaton et al. (2021). Training Peers to Ease Hospital Discharge: A Community–Clinical Partnership in Complex HIV Care [56].

## Data Availability

Data will be shared upon request to the first author.
